# A fast-flexible strategy based approach to solving employee scheduling problem considering soft work time

**DOI:** 10.1038/s41598-024-56745-4

**Published:** 2024-03-14

**Authors:** Kangxin Ma, Changchun Yang, Haojie Xu, Hexin Lv, Feng Hong

**Affiliations:** 1 School of Computer Science and Artificial Intelligence, Changzhou University, Changzhou, 213164 China; 2 School of Information Science and Technology, Zhejiang Shuren University, Hangzhou, 310015 China; 3 State Grid WenZhou Electric Power Supply Company, Wenzhou, 325035 China

**Keywords:** Employee scheduling, Soft work time, Pairwise-allocated strategy, Proficiency average matrix, Engineering, Electrical and electronic engineering

## Abstract

Employee scheduling aims to assign employees to shifts to satisfy daily workload and constraints. Some employee scheduling problems and their variants have been proven NP-hard, and a series of works have been done. However, the existing algorithms consider the fixed work time, which may cause plenty of overstaffing and understaffing phenomenons. Hence, this paper proposes a fast-flexible strategy based approach (FFS) to solve it. FFS introduces the idea of soft work time, which allows the work time of employees can be adjusted in a range. Based on this, we set the flextime strategy to decide the specific work time of each employee every day. Besides, FFS adopts a pairwise-allocated strategy and proficiency average matrix to boost its efficiency and effectiveness. Finally, the extensive experimental evaluation shows that FFS is more effective and efficient than the baselines for solving the employee scheduling problem considering soft work time.

## Introduction

Employee scheduling aims at assigning the right employees to the right shifts at the right time, for satisfying the constraints and achieving the optimization of goals^1^. It widely arises in real-life scenarios such as health care^2–4^, retail stores^5,6^, transportation^7^, job shops^8^ and call centers^9–11^. In most existing works, employees perform their duties according to the fixed period, which is composed of fixed start and end time points. However, the workload of different periods varies as time goes by, and employee scheduling considering fixed work time can not satisfy the varied workload well, and causes a lot of understaffing and overstaffing problems. In the following, we consider one representative motivation example.

### Motivation example (*call center*)

**Figure 1:**
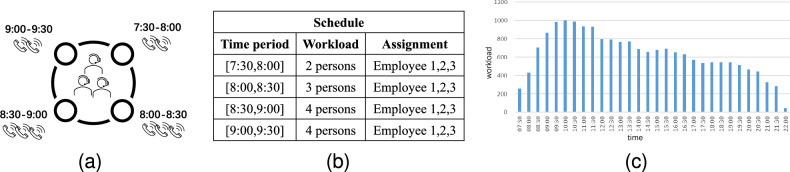
Illustration for motivation example.

In a call center, employees are assigned to shifts and serve the call arrivals as Fig. 1a shows. In Fig. 1a, three employees are assigned to the same shifts, suppose that the execution time of this shift is [7:30, 9:30], every 30 minutes is treated as a period and each one contains the number of call arrivals (treated as workload) as Fig. 1b shows. Combined with these two figures, employee scheduling considering fixed work time causes some understaffing and overstaffing problems. For example, the period [7:30, 8:00] requires 2 employees to satisfy its workload, but there are 3 employees, which is over the requirement of workload and causes the overstaffing problem. Besides, the period [9:00, 9:30] asks for 4 employees, while it is assigned to 3 employees, and thus the understaffing problem arises. Hence, employee scheduling considering fixed work time can not satisfy the varied workload well. The execution time of a normal shift usually contains several hours, and the corresponding workload of different periods is even more drastic as Fig. 1c shows, thus, the existing fixed work time scheduling causes plenty of overstaffing and understaffing phenomenons. In this paper, we introduce the idea of soft work time, which allows adjusting start and end time points. In real scenarios, there are several types of shifts with different execution times, and each one can adjust its start and end time points. When the number of assigned employees is over the requirement of workload, some employees can delay the start point of execution time or be earlier to end the work. Note that the adjustable periods exist at the start/end/meal point of the execution.

However, the existing studies suffer from one or more drawbacks in solving such employee scheduling problems.

1. The existing works rarely consider the soft work time, they stressed fixed work time.
2. Employee scheduling problem involves more than one optimization goal, and they may contain potential conflicts. that means the improvement of one goal may lead to the performance degradation of another. Taking the number of employees and output in manufacturing, enterprises ask for the least number of employees and the most output. Reducing the number of employees will decrease the output, and increasing the output asks for more employees. Many studies convert multiple goals to one single objective by empirically using different weights over different goals^9,12^. However, too much domain knowledge is needed in this way and the generated schedule may fail if the weights are changed, hence, this type of method usually takes a lot of time.
3. Some employee scheduling problems and their variants have been proven NP-hard^13^, traversing each potential schedule will cost prohibitive computation. Hence, there are plenty of pruning techniques such as sample averaging approximation (SAA)^14^, these techniques can effectively work in their problems. However, as far as we know, these problems rarely consider soft work time. Hence, these pruning techniques can not address our problem well.

In our work, we consider the soft work time. Specifically, we consider two types of soft work time, i.e. the number of consecutive working days and the working duration of one day. To address this issue, we propose FFS, a polynomial-time method to address it. Specifically, FFS is divided into four steps. In the first step, FFS proposes a scheduling-cycle-based hard constraints control mechanism, to decide the soft consecutive working days. In the second step, FFS uses the gradient descent projection to estimate the number of employees required for each shift, according to workload and work time of each sharing period. It can implement coarse-grained pruning of the search space. In the third step, based on the estimated number of employees in the second step, FFS uses the pairwise-allocated strategy to find the suitable employee combinations and establishes the proficiency average matrix to further boost its efficiency. Thus, we generate the feasible assignments for each day in the whole scheduling horizon. In the fourth step, according to the workload coverage of each period, a flextime strategy is proposed to decide the specific work time of each employee performing his assigned shift.

In a nutshell, the key contributions of our paper are listed below.

1. We present a polynomial-time solution to efficiently solve employee scheduling considering soft work time. It provides several strategies to effectively shrink the number of potential schedules, and fast find high-quality employee assignments.
2. We further consider two types of soft work time, and propose allocation strategies to decide the specific work time of each employee. It can alleviate the overstaffing and understaffing problems, and improve the quality of schedules.

The rest of this paper is organized as follows. Section "Preliminaries" introduces basic concepts and gives a formal definition of the problem. Section "Algorithm overview" elaborates on our approach FFS. Experimental results and our findings are reported in Sect. "Experiments". Section "Related work" reviews related work. Finally, Sect. "Conclusion" concludes the paper with some directions for future work.

## Preliminaries

In this section, we first present a series of decisions, i.e. scheduling horizon, shifts, and employees. Then we introduce the scheduling constraints. Finally, we define the optimization goals.

### Decisions

We start with the description of each decision.

#### Scheduling horizon

The scheduling horizon D is defined by

1 $$\begin{aligned} &D=\{d_1,d_2,\dots ,d_n\}\\&d_i=\{tp_i^1,tp_i^2,\dots ,tp_i^{\omega }\}\\ \end{aligned} $$

where $d_i$ $\in$ $D$ denotes a day. Each day has the same duration for work, and can be divided into $\omega$ same consecutive time periods, denoted by $\{tp_i^1,tp_i^2,\dots ,tp_i^{\omega }\}$. Each time period $tp_i^j$ $\in$ *TP* has a corresponding workload, denoted by $W\_tp_i^j$.

#### Shifts

 There are 5 types of shifts assigned to employees, each type of shift contains an execution time, they are denoted by,

2 $$\begin{aligned} &SFT_k^i=\{\tau |\tau =\{1,2,3,4,5,0\}\}\\&T_k^i=[tp_i^1,tp_i^{18}], T_{ks}^{i}=[tp_i^1,tp_i^{4}], T_{ke}^i=[tp_i^{15},tp_i^{18}],T_{km}^i=[tp_i^{9},tp_i^{11}] \; if\; SFT_k^i=1;\\&T_k^i=[tp_i^2,tp_i^{21}], T_{ks}^{i}=[tp_i^2,tp_i^{5}], T_{ke}^i=[tp_i^{18},tp_i^{21}],T_{km}^i=[tp_i^{9},tp_i^{11}]\; if\; SFT_k^i=2;\\&T_k^i=[tp_i^8,tp_i^{27}], T_{ks}^{i}=[tp_i^8,tp_i^{11}], T_{ke}^i=[tp_i^{15},tp_i^{18}],T_{km}^i=[tp_i^{20},tp_i^{22}]\; if\; SFT_k^i=3;\\&T_k^i=[tp_i^{13},tp_i^{30}], T_{ks}^{i}=[tp_i^13,tp_i^{16}], T_{ke}^i=[tp_i^{27},tp_i^{30}],T_{km}^i=[tp_i^{20},tp_i^{22}] \; if\; SFT_k^i=4;\\&T_k^i=[tp_i^5,tp_i^{7}]\cap [tp_i^{19},tp_i^{29}], \; if\; SFT_k^i=5; \end{aligned} $$

Where *i* is the $i^{th}$ day; *k* is the $k^{th}$ employee; $SFT_k^i$ is the assigned shift of $k^{th}$ employee for $i^{th}$ day; $\tau$ is the types of shifts which can be assigned to employees; in the $\{1,2,3,4,5,0\}$, $\{1,2,3,4,5\}$ is the shifts for working and $\{0\}$ is the rest day; $T_k^i$ is the execution time for $k^{th}$ employee for $i^{th}$ day; $T_{ks}^i$ is the start execution time for $k^{th}$ employee for $i^{th}$ day; $T_{ke}^i$ is the end execution time for $k^{th}$ employee for $i^{th}$ day; $T_{km}^i$ is the meal time for $k^{th}$ employee for $i^{th}$ day. One employee can be assigned to one shift at most. Each shift has an execution time, which contains a series of consecutive time periods. In addition, the execution time of each shift is unfixed. Specifically, the start and end time periods of execution time are selected in the first four time periods and the last four time periods.

#### Employees

 There are t employees, each one has a proficiency, and each time period has a total proficiency, which is denoted by,

3 $$\begin{aligned} &E=\{e_1,e_2,...e_k,...,e_t\}\\&P=\{p_k|e_k\in E\}\\&total\,p\_tp_i^j=\sum _{1}^{t} p_k, \; SFT_k^i\ne 0\; and\; tp_i^j\in T_k^i \end{aligned} $$

where *k* is the $k^{th}$ employee; $p_k$ is the proficiency of $k^{th}$ employee; $totalp\_tp_i^j$ is the total proficiency of time period $tp_i^j$ for the employee assigned to working shifts.

### Optimization objectives

(1) Average workload coverage *Ave_Coverage*. *Ave_Coverage* can be computed by the whole workload and total assigned employees’ proficiency (n days and each day $\omega$ time periods), which can be defined as below.

4 $$\begin{aligned} &min\,f1 = \left| 1-\frac{1}{n \times \omega }\times \sum _{i=1}^{n}\sum _{j=1}^{\omega }\frac{totalp\_tp_i^j}{W\_tp_i^j}\right| \\&totalp\_tp_i^j=\sum _{1}^{t} p_k, \; SFT_k^i\ne 0\; and\; tp_i^j\in T_k^i \end{aligned} $$

where $tp^j_i$ is the $j^{th}$ time period of $i^{th}$ day; $totalp\_tp_i^j$ is the number of total assigned employee proficiency for $tp^j_i$; $W\_tp_i^j$ is the workload of $tp^j_i$; n is the number of days in scheduling horizon; $\omega$ is the number of time periods in one day; *t* is the number of employees. No matter the average workload coverage of one day or one time period, it should be closest to 1. If coverage > 1, it means that the assigned proficiency is too much and more than the requirement of the workload, which causes the waste of employee proficiency. When coverage < 1, it means the assigned proficiency is too little and less than the requirement of the workload, where the workload can not be finished.

(2) Coverage fairness *Coverage_Fairness* can be computed by the whole workload and total assigned employees’ proficiency, which can be defined as below.

5 $$\begin{aligned} &min\, f2 = \biggr \{\frac{1}{\omega \times n}\times \sum _{i=1}^{n}\sum _{j=1}^{\omega }(\frac{totalp\_tp_i^j}{W\_tp_i^j}-Ave\_Coverage)^2\biggr \}^{1/2}\\&totalp\_tp_i^j=\sum _{1}^{t} p_k, \; SFT_k^i\ne 0\; and\; tp_i^j\in T_k^i \end{aligned} $$

where $tp^j_i$ is the $j^{th}$ time period of $i^{th}$ day; $totalp\_tp_i^j$ is the number of total assigned employee proficiency for $tp^j_i$; $W\_tp_i^j$ is the workload of $tp^j_i$; *Ave_Coverage* is the average workload coverage for n days, which can be computed by Eq. (2); n is the number of days in scheduling horizon; $\omega$ is the number of time periods in one day; *t* is the number of employees. Coverage fairness should be minimal, which means the coverage fluctuation of time periods.

### Constraints

(1) Each employee consecutively works max days at most, but no less than min days, which can be defined as below.

minimal day constraint:

6 $$\begin{aligned} &SFT_k^i = 0\; and\; SFT_k^{i+min+1}=0,\; if \; SFT_k^i \times SFT_k^{i+1}\times SFT_k^{i+2} \times \dots \times SFT_k^{i+min+1} = 0 \\&s.t.\; 0\le i\le n-min \end{aligned} $$

maximal day constraint:

7 $$\begin{aligned} &\; SFT_k^i \times SFT_k^{i+1} \times SFT_k^{i+1}\times \dots \times SFT_k^{i+max+1} \ne 0\\&s.t.\; 0\le i\le n-max \end{aligned} $$

(2) Each employee should have r rest days for the whole scheduling horizon and any two consecutive rest days are not allowed, which can be defined as below.

8 $$\begin{aligned} &\left\{ \begin{matrix} SFT_k^i \in R_k, &{} if\; SFT_k^i=0; \\ |R_k|=r&{}\\ SFT_k^i+SFT_k^{i+1}\ne 0 &{} \end{matrix}\right. \\ \end{aligned} $$

### Objective function

Traditional objective function gathers all objectives with weights, but the weights need more time to be adjusted. Hence, this paper used the TOPSIS to evaluate the indicated solutions without weights. More details are presented as our other work^15^.

9 $$\begin{aligned} max\{TOPSIS(-f1,-f2)\} \end{aligned}$$

Where function TOPSIS measures two optimization objectives to generate a score at the same time, here, the score is higher, the quality of the result is better, the score is lower the quality of the result is worse.

## Algorithm overview

To generate a feasible schedule with flexible work time, a naive way to address this problem is to traverse all the potential schedules, and select the best result among them as the final solution. However, such a method requires prohibitive computation consumption, since the number of potential schedules grows exponentially as the number of employees increases.

**Algorithm 1:**
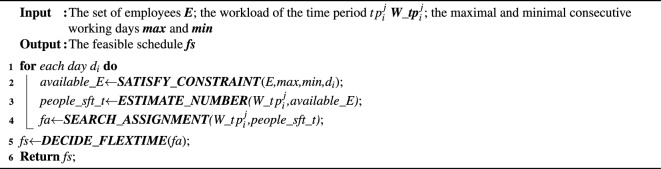
The overview of our approach.

Hence, we propose a fast-flextime strategy based approach (FFS) to efficiently search for a feasible schedule, which is a polynomial-time method. For ease of understanding the main idea of FFS, the pseudo-code of algorithm overview is presented in Algorithm 1. To be more specific, we generate the employee assignment over days (line 1), and the employee assignment of each day is generated by four modules, namely, satisfying constraints *SATISFY_CONSTRAINTS*() (line 2), estimating employee number *ESTIMATE_NUMBER*() (line 3), searching feasible assignments *SEARCH_ASSIGNMENT*() (line 4) and deciding flexible work time *DECIDE_FLEXTIME*() (line 5).

### Satisfying constraints

To ensure the availability of the generated schedule, *SATISFY_CONSTRAINTS*() is required to meet the hard constraints. Its pseudo-code is presented in Algorithm 2. *SATISFY_CONSTRAINTS*() takes a set of employees (*E*), the certain day ($d_i$) and the number of days in scheduling horizon (*D*) as input, and output is available employee set (*available_E*).

**Algorithm 2:**
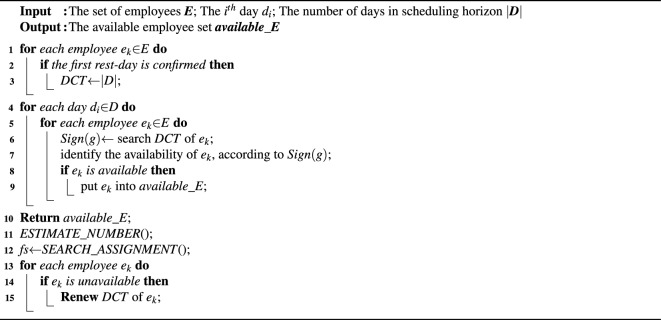
SATISFY_CONSTRAINT (*E*,$$d_i$$,|*D*|).

To achieve satisfying hard constraints, *SATISFY_HCONSTRAINT*() adopts the constraint control mechanism based on scheduling cycle (*SC*), which is composed of $\xi$ consecutive workdays and a rest day, e.g. the shift sequence (*sft_1*,*sft_2*, *rest-day*) contains 2 working days and 1 rest-day, which can compose a *SC*. The types of such *SC* are dependent on *max* and *min*. Since $H_2$ asks each employee works *max* days at most, but no less than *min* days, we get $\xi$ $\in$[*min*,*min*]. Suppose that *min*=2, *max*=4, the scheduling cycles contain three types, i.e. 2W+1R (2 workdays and 1 rest-day), 3W+1R (3 workdays and 1 rest-day) and 4W+1R (4 workdays and 1 rest-day).

**Figure 2:**
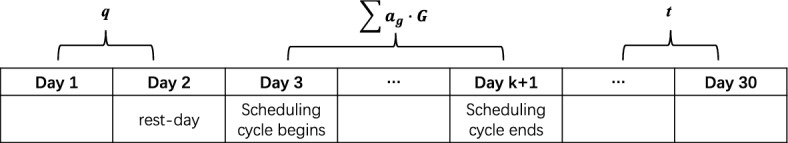
Classification of time points in DCT.

Then *SATISFY_CONSTRAINTS()* computes the DCT of each employee (line 3), which can be divided into 3 categories: the number of days before the first rest-day ***q***, the number of days in all types of scheduling cycles $\sum a_g \cdot G$, and the left days ***t***, as Fig. 2 shows.

Note that the first rest day of each employee does not belong to any type of scheduling cycle, and $H_3$ asks for *r* rest day for each employee in the scheduling horizon. Hence, the total number of scheduling cycles for each employee is $r-1$. Besides, as for *t*, due to the minimal number of consecutive workdays are set to *min* days, as long as $t\le min$, employees will be assigned to shifts in these *t* days, and the total number of rest-days for each employee are fixed to *r* days.

The formal for computing DCT is as below.

10 $$\begin{aligned} \begin{aligned} q+\sum _{\begin{array}{c} g\in Z^+\\ g\in [min,max] \end{array}}&a_g\times g+t=n\\&s.t.\ q,t\in Z^+\\ {}&0\le q\le max;\\ {}&0\le t\le min;\\ {}&\sum a_g=r-1. \end{aligned} \end{aligned}$$

According to the above operations, *SATISFY_HCONSTRAINT*() module computes DCT of each employee. Since the serial number of rest days is different for each employee, their DCT is different. For each employee $e_k$ $\in$*E*, *SATISFY_HCONSTRAINT*() will check the return value (*sign(g)*), and identify the availability of employees. Here, *SATISFY_HCONSTRAINT*() traverses previous *max* shifts for each employee, and the number of workdays in this shift sequence is marked as *NUM*, which is treated as a trigger to search for suitable SC. There may exist three situations:

1. $NUM<min$, all employees are available;
2. $min\le NUM\le max$ and $NUM\in sign(g)$ and $\forall g \in sign(g)\le NUM$, there exists the scheduling cycle whose number of work days equals to *NUM*, but that whose number of work days exceeds *NUM* does not exist. Thus, $e_k$ is unavailable;
3. $min\le NUM\le max$ and $NUM\in sign(g)$ and $\exists g \in sign(g)>NUM$, compared to situation 2, situation 3 exists that whose number of work days exceeds *NUM*. Thus, $e_k$ is available.

### Estimating employee number

The available employees of $d_i$ are generated by *SATISFY_HCONSTRAINT*(). Suppose that the number of available employees is $\beta$, given five types of shifts (defined in Para.1 Page 3), if we assign directly available employees to shifts, each employee can be assigned to anyone in these five types of shifts, and thus, the number of potential assignments is $5^\beta$, i.e. exponential. To avoid such a situation, we invoke the procedure *ESTIMATE_NUMBER*() (as algorithm 3 shows), which takes available employees and the workload of each time period in $d_i$ as inputs, and the output is the estimated number of employees required for each shift of $d_i$. In the following, we give an example to show how it works, combined with Algorithm 3.

**Algorithm 3:**
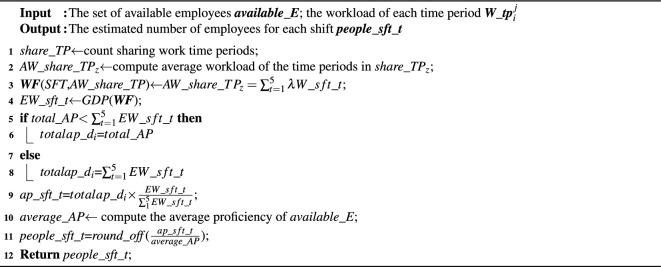
Estimate_number(*available_E*,*W_tp*$$_\textit{i}^\textit{j}$$).

**Figure 3:**
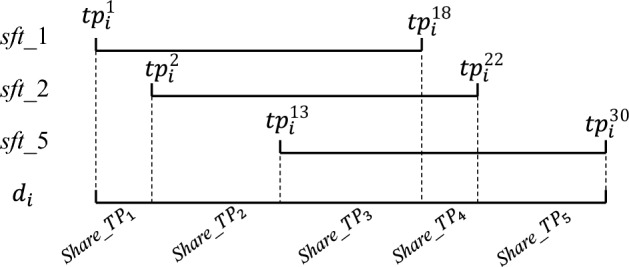
Example of computing sharing work time among shifts.

First, according to the work time of each shift, we count the sharing work time periods *share_TP* among different shifts. For instance, given three shifts of $d_i$, i.e. *sft_1*, *sft_2*, *sft_5* and their work time are [$tp_i^1,tp_i^{18}$], [$tp_i^2,tp_i^{22}$] and [$tp_i^{13},tp_i^{30}$], respectively, These work time are divided into different share time periods as Fig. 3 shows (line 1), e.g. *share_TP*$_\textit{1}$=[$tp_i^1,tp_i^2$), *share_TP*$_\textit{2}$=[$tp_i^2,tp_i^{13}$). Then we compute the average workload *AW_share_TP*$_\textit{z}$ of each sharing work time period *share_TP*$_\textit{z}$ (line 2). Next, we establish the workload function as follows (line 3) to estimate the workload of each shift of $d_i$.

11 $$\begin{aligned} &AW\_share\_TP_z=\sum _{t=1}^{5}\lambda W\_sft\_t\\&s.t.\quad \lambda =\left\{ \begin{matrix} 0, &{} share\_TP_z\notin sft\_t; \\ 1,&{} share\_TP_z\in sft\_t; \end{matrix}\right. \\ \end{aligned} $$

Where *AW_share_TP*$_z$ represents the average workload of *share_TP*$_\textit{z}$, $\lambda$ is a parameter for checking whether *share_TP*$_\textit{z}$ belongs to the work time of the corresponding shift, *W_sft_t* is the workload of *sft_t*.

Note that if *share_TP*$_\textit{z}$ belongs to the work time of *sft_t*, $\lambda =1$; Otherwise, $\lambda =0$. Thus, Eq. (11) represents that the average workload of *share_TP*$_z$ is composed of the workload of each shift. However, estimating the workload of each shift by solving Eq. (11) is so strict that there may not exist a feasible solution, since the number of sharing work time periods is regularly larger than the type of shifts ($z\ge t$), Eq. (11) is an overdetermined function^16^. Hence, we adopt the projected gradient methods to generate the solution of Eq. (11), where an error $\Delta b$ is introduced. Eq. (11) is converted into $\Delta b$ + $AW\_share\_TP_z=\sum _{t=1}^{5}\lambda W\_sft\_t$, where $\Delta b$ should follow that (1) the variance of any two sub-errors is the same, and (2) any two sub-errors are independent^16^. For easing to remember, we denote $\lambda W\_sft\_t$ and $AW\_share\_TP_z$ by *Ax* and *B*, where *A* is a matrix composed of $\lambda$, *x* is the estimated workload for each shift of $d_i$ and *B* denotes the workload of each sharing time period. Thus, we get Eq. (12) from Eq. (11).

12 $$\begin{aligned} ||\Delta b||=||Ax-B|| \end{aligned}$$

Note that we vary the value of *x* and make the error $\Delta b$ as small as possible. Thus, we use the least square method to reach this goal, which is achieved as below.

13 $$\begin{aligned} \min _{x} ||\Delta b||^2=||Ax-B||_2^2=(Ax-B)^T(Ax-B) \end{aligned}$$

where $||\Delta b||$ reaches the minimum, *x* is the feasible solution. To get the solution *x*, we introduce the projected gradient method, which is defined below.

14 $$\begin{aligned} \begin{aligned} x_{k+1}= P_C(x_k&-\mu \nabla f(x_k))\\ s.t.\ P_C(x)=(x)^+\Leftrightarrow&[(x)^+]_i=max\{x_i,0\} \end{aligned} \end{aligned}$$

where $P_C(x)$ denotes the projection operator, *C* represents the solution space for *x*, $\mu$ is the step length and $\nabla f(x_k)$ is the gradient vector. The workload of each sharing time period must be greater than 0, hence $C={\mathbb {R}}_+^n$. In this way, we estimate the workload for each shift *EW_sft_t* (line 4).

However, the proficiencies of all available employees may not satisfy the workloads of shifts. Hence, we need to confirm the total proficiency by *total_AP* and $\sum$*EW_sft_t* as below.

15 $$\begin{aligned} &totalap\_d_i=\\&\left\{ \begin{aligned}&total\_AP,\quad total\_AP\le \sum _{1}^{5} EW\_sft\_t; \\&\sum _{t=1}^{5} EW\_sft\_t, \quad total\_AP \ge \sum _{1}^{5} EW\_sft\_t. \\ \end{aligned} \right. \end{aligned} $$

where *total_AP* is the total proficiency of all available employees, and $\sum _{1}^{5} EW\_sft\_t$ is the total workload of all shifts for $d_i$.

When $total\_AP\le \sum _{1}^{5} EW\_sft\_t$, all the available employees can be assigned to shifts and the number of available proficiency *totalap_d*$_\textit{i}$ is set to *total_AP* (lines 5-7). When $total\_AP\ge \sum _{1}^{5} EW\_sft\_t$, not all available employees can be assigned to shifts for satisfying $S_1$, thus the number of available proficiency *totalap_*$\textit{d}_\textit{i}$ is set to $\sum _{1}^{5} EW\_sft\_t$ (lines 7-8).

Based on these, we estimate the number of proficiency *ap_sft_t* for each shift according to the ratios among *EW_sft_t* (line 9), and compute the estimated number of employees required for each shift *people_sft_t* by *ap_sft_t* and the average proficiency of all available employees *average_AP* (lines 10-11), then return *people_sft_t* (line 12).

### Searching feasible schedule

**Figure 4:**
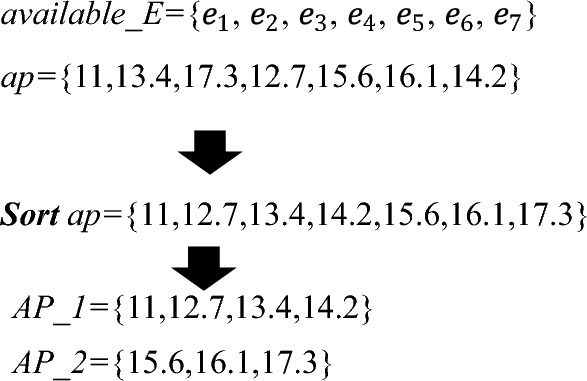
Dividing the proficiencies set into two subsets.

According to the estimated number of employees for each shift of $d_i$, *SEARCH_ASSIGNMENT*() requires to select employees and assign them to the corresponding shifts, which should follow the principle that the average proficiency of employee combinations for each shift should be maximally close to *average_AP*, since the closer to *average_AP* the average proficiency of employee combinations is, the smaller the values of $|1-Ave\_Coverage|$ and $Coverage\_Fairness$ are. Hence, we adopt the pairwise-allocated strategy to achieve this goal, and introduce the proficiency average matrix to boost its efficiency. In the sequel, combined with the procedure *SEARCH_ASSIGNMENT*() as Algorithm 4 shows, we present more details with the following example as Fig. 4 shows, where there are 7 available employees and their proficiencies are 11, 13.4, 17.3, 12.7, 15.6, 16.1, 14.2, *SEARCH_ASSIGNMENT*() assigns the estimated number of employees to the corresponding shifts.

**Algorithm 4:**
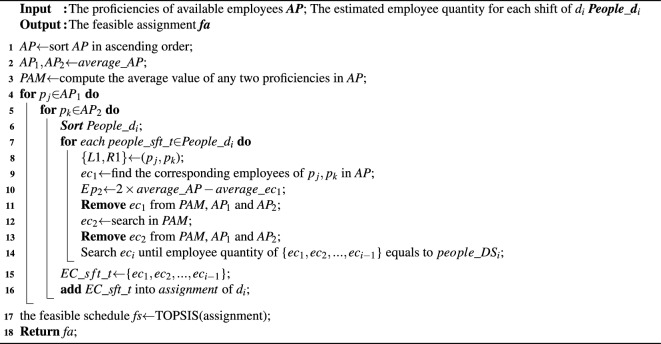
*SEARCH_ASSIGNMENTS*(*available_E*,*People_*$\textit{d}_\textit{i}$).

**Figure 5:**
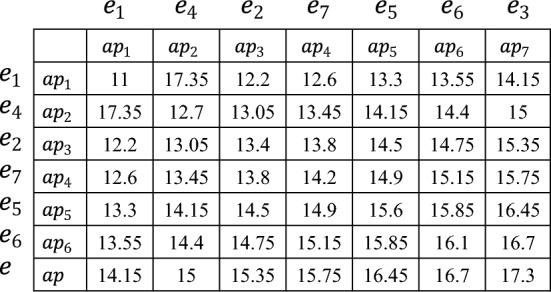
The example of establishing the average proficiency matrix.

First, the proficiencies of all available employees are sorted in ascending order (line 1), and they are divided into two subsets (i.e. *AP_1* and *AP_2*) according to *average_AP* (line 2), where *AP_1*={11, 12.7, 13.4, 14.2} and *AP_2*={15.6, 16.1, 17.3}. Then we compute the available value of any two proficiency in AP to establish the proficiency average matrix (*PAM*) as Fig. 5 shows (line 3), which is defined as a square matrix of *available_E*. Next, we take the first proficiencies of*AP_1* and *AP_2*, denoted by {*L*1,*R1*}={11, 15.6} (lines 4-6). Subsequently, we sort *People_sft_t* in an ascending order, which stores the estimated number of each shift, and starts with the least number of shifts (lines 6-7).

**Figure 6:**
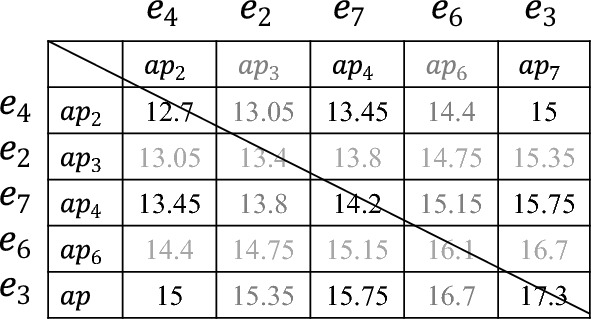
The example of searching $ec_2$.

Subsequently, we get the corresponding employee combination *ec*$_\textit{1}$={$e_1,e_5$} (lines 8-9), and its the average proficiency *average_ec*$_\textit{1}$ (=$\frac{11+15.6}{2}=13.3$) is a trigger to find the next employee combination $ec_2$. To ensure the average proficiency of $ec_1$ and $ec_2$ be as close to *average_AP* as possible, the expected average proficiency of $ec_2$ $Ep_2=2\times average\_AP-average\_ec_1=15.4$ (line 10). Pairwise-allocated strategy searches the value that is closest to *Ep*$_2$ (=15.4) in this *PAM*. It is worth noting that for satisfying the hard constraint $H_1$ (i.e. *each employee is assigned to at most one shift per day*), the employees in $ec_2$ are selected from the available employees except $e_1$ and $e_5$ (lines 11, 13) as Fig. 6 shows, and based on this, $ec_2=\{e_6,e_7\}$, since the average proficiency of $e_6$ and $e_7$ is 15.15, which is closet to $Ep_2$ (= 15.4 line 10) (line 12).

In the sequel, the pairwise-allocated strategy computes the expected average proficiency of $ec_i$ ($i\ge 3$) and locates $ec_i$ in the same way until the total number of employees in the selected groups equals the estimated number (line 14). Note that, if the estimated number of employees is odd, the last employee is treated as a group, whose selection way also follows the principle that the average proficiency of this group should be maximally close to the expected average proficiency (lines 15-16).

Then, we select the first proficiency in $AP\_1$ and the second proficiency $AP\_2$, denoted by {*L*1,*R*2}={11, 16.1}, and the corresponding employee group $ec_1=\{e_1,e_6\}$ is utilized to generate the next candidate assignment until all combinations of proficiency between *AP_1* and *AP_2* are listed.

In the end, we introduce the TOPSIS (Technique for Order Preference by Similarity to an Ideal Solution, TOPSIS^17^) to evaluate the selected assignments, where each one will be scored by TOPSIS. The assignment with the highest score is treated as the feasible schedule (line 17). Traditional multiple-objective optimization algorithms usually use the linear weighted method, which uses weights to transform different optimization objectives into one. However, weight setting requires a large amount of domain knowledge and expert experience, and needs a lot of time to choose suitable weights. Compared with these, TOPSIS rarely considers the weights among the optimization objectives, and this is the reason for choosing TOPSIS. The more details are explained in reference^17^.

### Deciding flexible work time

To maximally satisfy the workload of each time period for each day, the assignments generated from Sect. "Searching feasible schedule" require deciding the flexible work time of each shift. To achieve this goal, we invoke the procedure *DECIDE_FLEXTIME*() (as Algorithm 5 shows), which takes the feasible assignment (*fa*) as the input, and the output is the feasible schedule with flexible work time (*fs*).

First, according to the feasible assignment *fa*, we compute the total average proficiency of *fa* (lines 1-2). Based on these, we compute the coverage of each time period for the assignment of $d_i$, and get the employee sets for each shift, i.e. *E_sft_t* (lines 3-5). Next, we get the corresponding proficiency sets for these employee sets, and sort the proficiencies of them in ascending order (line 6).

Then we need to confirm the flexible work time of each shift (lines 7-8), which is divided into three categories, i.e. the start work time periods, the end work time periods and the meal break, hence we propose three corresponding strategies to deal with these.

**Algorithm 5:**
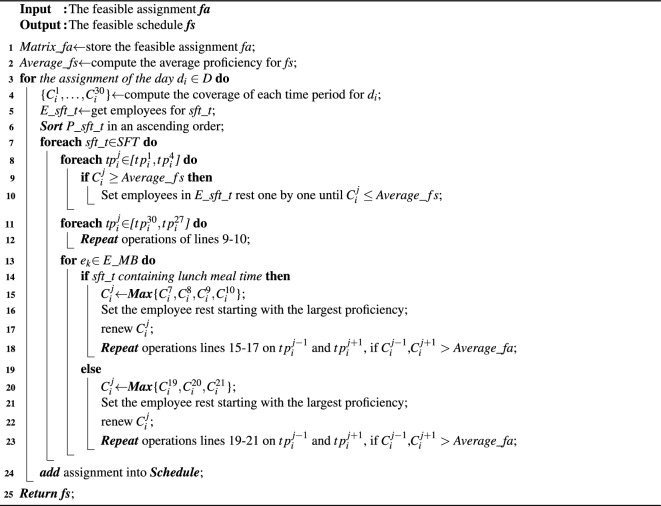
*DECIDE_FLEXTIME*(*available_E*,*ap*,*fs*).

***Strategy 1:*** (The flexible start work time periods) The time periods for flexible start work are [$tp_i^1,tp_i^4$], we need to compare the coverages of these time periods with *Average_fs* in turn. For each coverage $C_i^j$ of the time period $tp_i^j$ $\in$ [$tp_i^1,tp_i^4$], if $C_i^j\ge Average\_fs$, we remove the proficiency in *P_sft_t* in turn. The reason is that the workloads of these time periods are small, and removing the proficiency from smallest to largest will make coverage maximally get close to *Average_fa*, on the premise of decreasing the influence on the coverage of these time periods (lines 9-10). In addition, if $e_k$ is working in the time period $tp_i^{j-1}$, he can not be set to rest in $tp_i^j$.

***Strategy 2:*** (flexible end work time periods) The time periods for flexible end work are [$tp_i^{27},tp_i^{30}$] and we compare coverages of these time periods with *Average_fs* in decreasing order in turn, then we do the same operations (lines 11-12) in strategy 1. Similarly, if $e_k$ is working in the time period $tp_i^{j+1}$, he can not be set to rest in $tp_i^j$.

***Strategy 3:*** (flexible meal break) Different from the start and end work time periods, each employee must have a meal break, when he can have lunch or supper. In addition, the meal break is divided into lunch meal time and dinner meal time, hence we should identify which one belongs to the shift *sft_t* of $e_k$ (line 13-14,18). Based on these, if *sft_t* contains the lunch meal time, we select the higher coverage ratio of the time periods $tp_i^7$, $tp_i^8$, $tp_i^9$ and $tp_i^{10}$, denoted by $c_i^j$ (line 15). Next, we set employees in *E_sft_t* starting with the largest proficiency, the corresponding proficiency is removed, and $C_i^j$ is renewed (lines 16-17). Then we check the adjacent time periods of $tp_i^j$ to have a longer meal time (line 18). If *sft_t* contains the dinner meal time, we do the same operations on the time periods of the dinner meal, i.e, [$tp_i^{19},tp_i^{21}$] (lines 19-23).

Thus, each employee assigned to shifts of $d_i$ has flexible work time and meal break, and is added to the assignment of $d_i$ in the feasible schedule (*fs*) (line 22). Finally, when the assignments of all days in the scheduling horizon are performed *DECIDE_FLEXTIME*(), *fs* is treated as the feasible schedule with flexible work time and returned (line 23).

### Discussion

This subsection discusses each module of our approach in terms of time complexities by the book with the title “New Generation Computer algorithm”^18^. Then, the existing algorithms are compared to our algorithm with time complexity.

*SATISFY_CONSTRAINTS* This module is composed of two parts: dynamic combination table (DCT) computation and DCT query. Since the dynamic combination tablets can be generated in advance, this part of time complexity is negligible. In the part of the DCT query, due to the number of types of scheduling cycles being constant, hence its time complexity is *O*(1).

*ESTIMATED_NUMBER* This module is composed of a sequential structure, where the highest time complexity is the Gradient Descent Projection (GDP). Although its time complexity is hard to evaluate, this computation can be processed in advance. As for other operations in this module, the computation is constant and the time complexity is *O*(1).

*SEARCH_ASSIGNMENT* In this module, the available employees are divided into two sub-sets. In the worst case, the number of first employee combinations is $(\frac{n}{2})^2$. The next employee combination will be selected by whose proficiency can make the first one’s proficiency closest to the average proficiency *O*(1). Thus, the time complexity of this module is $O(n^2)$.

*DECIDE_FLEXTIME* This module decides the work time of each employee in sequential order. Hence, its time complexity is *O*(n).

Based on these, the time complexity of our approach is *O*($m\cdot n^2$), where *m* is the number of days on the scheduling horizon.

Our problem is a new one, the heuristic algorithm is designed for a specific problem, hence the existing heuristic algorithm is unsuitable for our problem, only general algorithms such as meta-heuristic algorithms can adapt to our problem. However, due to randomness of the generated results, the meta-heuristic algorithm (NSGA-II^19^, IPSO^20^, PICEA-g^21^, MOEAD^9^ and GF^22^) are required to run multiple times for deciding final results with rather high quality. Besides, they usually generate initial individuals and adopt evolutionary mechanisms to generate new individuals, then compare them to choose the better ones. Due to the mechanism of choosing, their time complexity is different. NSGA-II and MOEAD are $O(\beta \cdot n^2)$ and $O(\beta nT)$, where $\beta$ denotes the number of individuals of one generation, and *T* is the number of neighborhoods. IPSO is *O*(*n*!), PICEA-g is $O(n^3)$. GF is a novel general framework, which gathers the existing meta-heuristic algorithms whose time complexity ranges from $O(\beta nT)$ to *O*(*n*!). As for the MILP, we use the Gurobi solver 9.1 and the solution is a branch and bound method, the time complexity is *O*(*n*!).

In general, the time complexity of our algorithm is less than others.

## Experiments

In this section, we experimentally evaluate the efficiency and effectiveness of our proposed solution *FFS* against the state-of-the-art. We implement our algorithm in Python, and adopt the Python implementations of all competitors based on the following methods: Mixed-Integer Linear Programming (MILP^12^), Improved Particle Swam Optimization (IPSO^20^), A Fast and Elitist Multiobjective Genetic Algorithm (NSGA-II^19^), the Preference-inspired Co-evolutionary Algorithm Using Goal Vectors (PICEA-g^21^), Multi-objective Evolutionary Algorithm based Decomposition (MOEAD^9^) and a general multi-objective algorithm framework (GF^22^), which are listed in Table 1. The MILP adopts Gurobi solver 9.1^23^ to generate solutions. NSGA-II^19^, MOEAD^9^ and PICEA-g^21^ are three multi-objective evolutionary algorithms (MOEAs), and MILP^12^ belongs to the mathematical methods, the IPSO^20^ is the heuristic algorithms, and the GF^22^ is one of novel general framework for solving multi-objective optimization problems.

**Table 1:** The data sets used in experiments.

Methods	Category	Year
NSGA-II^19^	Meta-Heuristic	2021
MOEAD^9^	Meta-Heuristic	2020
MILP^12^	Mathematical	2020
IPSO^20^	Meta-Heuristic	2020
PICEA-g^21^	MOEA	2021
GF^22^	Meta-Heuristic	2020

Besides, to compare the performance of *FFS* and five methods with the considerations of fairness and accuracy, we (1) report the response time of each method by generating the same feasible schedule results, and (2) report the TOPSIS score of each method under the same response time. all evaluations in this section are performed based on a mixture of real and synthetic data sets. The real part is provided by the call center of China Telecom company, which is the call arrivals of six months from July 2020 to Dec. 2020. The synthetic part is the employees, which are synthesized from the real employees of a call center in China Telecom company. Both of these parts are listed in Table 2, where the number of employees is the real-life data. We synthesize five employee sets for each month, whose number of employees are 40, 60, 80, 100 and 120, respectively. We synthesize these employee sets by randomly choosing part of employees in real life as the added or reduced employees. In addition, each employee in these employee sets has a proficiency. Note that each experiment runs 10 times by randomly choosing the corresponding quantity of employees, and reports the average result. All the experiments are conducted on a server machine with an Intel Intel(R) Xeon(R) CPU E5-2637 3.50 GHz processor and 8GB RAM, running Windows 10 with Python 3.8.

**Table 2:** The data sets used in experiments.

Datasets	Days	Call arrivals	Employee number
July 2020	30	37,691	81
Aug. 2020	31	38,037	60
Sept. 2020	31	35,991	76
Oct. 2020	30	37,110	70
Nov. 2020	31	38,133	86
Dec. 2020	30	36,510	79

### Experiment setting

We totally set 6 sets of experiments to evaluate the performance of *FFS* and five alternatives, the parameters in each experiment are illustrated in Table 3, where the same quality means that five alternatives aim at generating a schedule with the quality same to that of *FFS* generating and report their response time, same run-time means that their response time is set to be same to that of *FFS* generating a schedule and report the quality of their schedules. **EXP1** to **EXP4** evaluate the overall performance difference among *FFS* and five alternatives by varying the number of employees and datasets. **EXP5** and **EXP6** evaluate the internal performance difference by removing the flextime-strategy, pairwise-allocated strategy and proficiency in turn.

**Table 3:** The parameters used in experiments.

EXPs	Data sets	Employee quantity	Same run-time	Same quality
EXP1	–	80		$\checkmark$
EXP2	–	80	$\checkmark$	
EXP3	Oct.	80		$\checkmark$
EXP4	Oct.	80	$\checkmark$	
EXP5	Oct.	–		$\checkmark$
EXP6	Oct.	–	$\checkmark$	
EXP7	–	80		$\checkmark$
EXP8	Oct.	80		$\checkmark$

### Overall performance

#### EXP 1: Search efficiency

**Figure 7:**
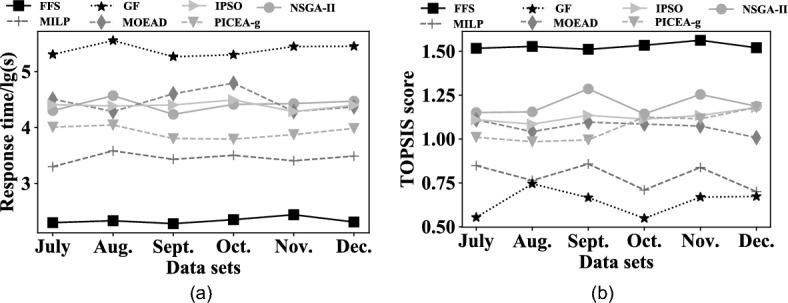
Overall effectiveness and efficiency with different data sets.

The first set of experiments verifies the performance of *FFS* by varying datasets, compared with the other six alternative methods. The result is shown in Fig. 7a. The first observation is that *FFS* has the shortest response time in all cases, with MILP, GF and NSGA-II in the second place, and MOEAD, PICEA-g and IPSO are the worst. Specifically, *FFS* outperforms MILP, GF and NSGA-II by one order of magnitude, and is faster than PICEA-g, IPSO and MOEAD two orders of magnitudes. The reason is that MILP needs to consider all the potential assignments, and even if adopting a series of fast computing sub-algorithms such as the simplicissimum method, MILP remains to be time-consuming. GF adopts the universe methods to solve this problem, but they lack of optimization strategy for our problem. As for NSGA-II, it adopts a fast non-dominated sorted strategy to speed up the convergence of solutions. MOEAD, PICEA-g, and IPSO require enough generation operations to get the feasible solutions, due to their random nature of query strategies; while *FFS* adopts the pairwise-allocated strategy to effectively shrink the number of potential assignments, which makes the feasible assignment query execute in a small solution space. The second observation is that *FFS* achieves the most stable performance and MOEAD fluctuates most greatly. The reason is that *FFS* effectively reduces the number of potentially feasible assignments, owing to pairwise-allocated strategy. While MOEAD requires the operations of mutation and crossover to generate the new assignments, and select ones with the quality higher than old assignments. However, the operations of mutation and crossover contain the nature of randomness, which results in the instability of newly generated solutions.

#### EXP 2: Search effectiveness on running the same time

 EXP 2 runs under the condition of running the same time and reports the TOPSIS score of each method as illustrated in Fig. 7b. It is seen that when changing the datasets, the TOPSIS score of *FFS* changes slightly, and gets the highest TOPSIS score. It is because, the flextime strategy of *FFS* according to the coverage ratio of each time period changes the work time of each employee, which follows the principle that each employee should have *r* rest days for each month and can not be assigned to rest day for two consecutive days. Thus, it ensures that two optimization goals (i.e. *Ave_Coverage* and *Coverage_Fairness*) can be closer to the optimal values. In addition, pairwise-allocated strategy in *FFS* selects suitable employee combinations according to the soft constraint $S_1$, and assigns them to the corresponding shifts. Hence, the TOPSIS score performs best. As for the NSGA-II, MOEAD and PICEA-g, these MOEAs usually require a large number of generations to ensure the quality of their solutions, but the time cost of this experiment is little, which limits the number of generations and the solutions of MOEAs can not be guaranteed to be high-quality. The MILP also faces a similar situation, which considers all the potential schedules and requires enough computations to support its search sub-algorithms, but the limited time cost weakens the quality of its solution. As for the IPSO, it is easy to fall into local-optimal status, hence, when the first solution is high-quality, it will get some better solutions than MOEAD, NSGA-II, MILP and PICEA-g. However, when the quality of the initial solution is low, it may have low-quality solutions in the final. All of these deeply influence the quality of the generated schedule, and lead to that the quality of solutions from our approach is superior to that of others.

#### EXP 3: Effect of the number of employees on search efficiency

**Figure 8:**
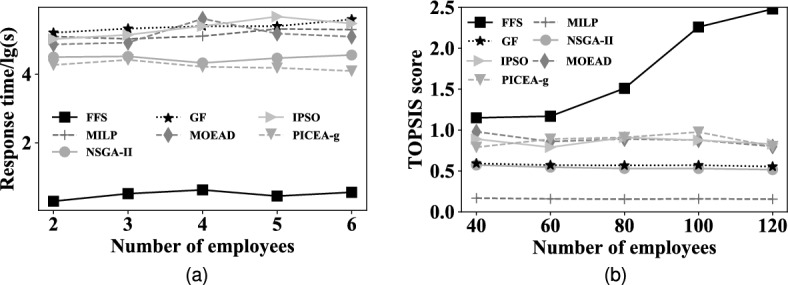
Overall effectiveness and efficiency with the number of employees.

 The third set of experiments evaluates the impact of the number of employees on search efficiency. The result is depicted in Fig. 8a. The first observation is that the response time of *FFS* slightly increases as the number of employees grows. It is because that, for *FFS*, the search space shrunken by pairwise-allocated strategy gets larger with the increasing number of employees, and *FFS* spends more time searching the suitable employee groups. The second observation is that the time cost of MILP and GF increases as the number of employees grows. The reason is that the number of potential feasible schedules increases exponentially for MILP and GF, although they contain a series of pruning techniques to reduce the search space, it remains to be pretty large and the growth of employee number adds to their response time. The third observation is that the response time of MOEAD, NSGA-II and PICEA-g fluctuates with the increase in the number of employees. The reason is that, they randomly initialize individuals, and generate the feasible schedule based on the search strategy with the nature of randomness, which leads to unstably of their generated schedules. To reach the quality of a fixed schedule, they have to spend more generations to find a suitable schedule, and are presented in the fluctuation of response time. As for IPSO, it is easy to fall into local-optimal, the time cost of running one is pretty short, but the quality of the generated schedule can not reach the fixed schedule, it will run again until it does. Thus, the total time cost is comparatively higher than others.

#### EXP 4: Effect of the number of employees on search effectiveness

Figure 8b shows the result of each method by varying the number of employees. It is observed that the TOPSIS score of *FFS* increases as the number of employees grows. It is because more employees mean more potential employee combinations, and thus, there is a higher possibility for *FFS* selecting the employee groups whose proficiency is nearest to the workload of shifts. Hence, the TOPSIS of FFS will increase with the number of employees growing. However, since the time cost is limited to that of FFS costing and it is too short, all alternatives’ query strategies are time-consuming, which results in a low number of generations and computations for MOEAs, IPSO and MILP. Thus, their generated schedules are of low quality. In view of these, the TOPSIS score of *FFS* is the highest in all cases.

### Internal performance

#### EXP 5: Internal performance vs. different datasets

**Figure 9:**
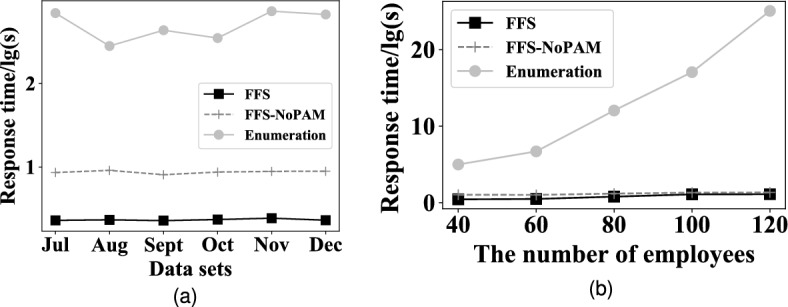
Overall effectiveness and efficiency with the number of employees.

The fifth set of experiments evaluates the internal impact of the performance of pairwise-allocated *PA* strategy and proficiency average matrix *PAM* by varying the datasets. We compare *FFS* with five alternative methods, i.e. *FFS-NoFlextime*, *FFS-NoPAM* and *Enumeration*, respectively. *FFS-NoPAM* removes the Average Proficiency Matrix *PAM*, and *Enumeration* enumerates all potential schedules. The result is illustrated in Fig. 9a. It is observed that *FFS* is faster than *FFS-NoPAM* and *Enumeration* on all datasets. In particular, *FFS* is faster than *FFS-NoPAM* by two orders of magnitudes, and outperforms *Enumeration* by 3 orders of magnitude in average, respectively. This is because, compared to *Enumeration*, *FFS* and *FFS-NoPAM* contain *PA*, which greatly reduces the number of potential schedules. This indicates that *PA* effectively shrinks the search range and improves efficiency. In addition, *FFS* adopts the proficiency average matrix (*PAM*) to boost the efficiency, and based on *PAM*, *FFS* outperforms *FFS-NoPAM* one order of magnitude, which indicates that *PAM* further improve the efficiency of search.

#### EXP 6: Internal performance vs. the number of employees

The sixth set of experiments explores the internal effect for *FFS* by varying the number of employees. The result is plotted in Fig. 9b. The first observation is that the response time of *Enumeration* is exponential, the reason is that the number of employee assignments grows exponentially as the number of employees increases, and the corresponding response time for *Enumeration* generating a schedule presents exponentially. The second observation is that the response time of *FFS* and *FFS-NoPAM* still remains low and stable, the reason lies in two aspects: first, they pre-estimate the number of employees for each shift of each day for pre-pruning a large number of potential schedules, which provides a pretty small range for searching the feasible schedule; second, they adopt the pairwise-allocated strategy to assign employees to shifts, where they only need few average proficiency computations instead of computing all employee combinations. The third observation is that the response time of *FFS* is less than that of *FFS-NoPAM*. The reason is that, *FFS* uses the proficiency average matrix to boost the efficiency of the pairwise-allocated strategy. *PAM* provides the average proficiency of all employees, which prunes the process of computing average proficiency among employees, and *PA* selects the suitable employee group with only a few computations.

**Figure 10:**
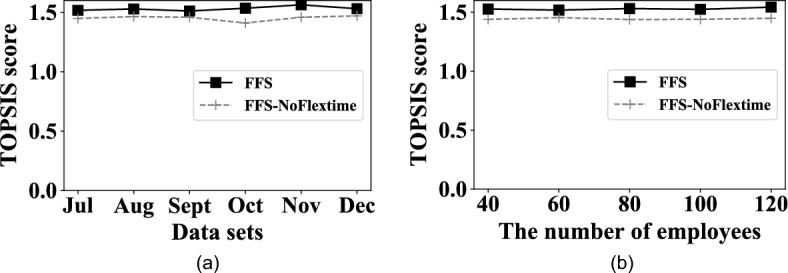
TOPSIS score of *FFS* by varying parameters.

#### **EXP 7: Flextime strategy vs. datasets.**

EXP 7 aims to explore the impact of the performance of flextime strategy on different datasets. The result is shown in Fig. 10a. It is seen that *FFS* has a higher TOPSIS score than *FFS-NOFlextime*. The reason is that the flextime strategy sets the flexible work time for each employee, which makes the assigned proficiency satisfy the workloads of different time periods in a fine-grained way. Then more satisfying workloads will present with higher TOPSIS scores.

#### EXP 8: Flextime strategy vs. the number of employees

The eighth set of experiments aims to explore the impact of the performance of the flextime strategy by varying the number of employees. The result is shown in Fig. 10b. Similar to EXP7, *FFS* has a better TOPSIS score than *FFS-NoFlextime*, and it is because that *FFS* adopts the flextime strategy to adjust the work time of employees for each day, the understaffing and overstaffing phenomenons have been improved.

## Related work

Employee scheduling problem is of significant importance in industries, such as healthcare, retail, and manufacturing. It made a great deal of progress in the past decades, and can be classified into three categories.

The first category is the mathematical methods, which model their employee scheduling problems and adopt open solvers such as LP^24,25^, IP^26,27^ and MIP^28^ solvers to generate feasible schedules. Basán et al.^29^ proposed a novel MILP-based decomposition method, for solving employee scheduling problems arising in manufacturing environments. However, this method requires a large amount of domain knowledge to model the problem. Meng et al.^12^ proposed four mixed integer linear programming (MILP) models as well as a constraint programming (CP) model to address the distributed flexible job shop scheduling problem with minimizing optimization goals. However, these works stressed the global result of the optimization objectives, but ignored the balance between the local result of each optimization objective on each day. Lunardi et al.^30^ present mixed integer linear programming and constraint programming models to address a flexible job shop scheduling problem with sequence flexibility in which precedence constraints among operations of a job. Although this work is performed well on small, medium, and large-sized instances, it generates the schedule with a one-day scheduling horizon, which arises in certain scenarios. A longer scheduling horizon (i.e. a week, a month, or longer) is a more regular phenomenon for most scenarios, and it means more difficult challenges such as temporal constraints. Our approach sets the hard constraints for these temporal constraints, and adopts a series of strategies to address the employee scheduling problem effectively and efficiently.

Although this category of the method has high effectiveness, a large amount of computation leads to low efficiency and high responding time. These methods do not provide the allocated strategy and search strategy as FFS does, and limit themselves to similar trips or other mathematical methods.

The second category is the meta-heuristic algorithm (MHA), which is one type of general algorithm and is suitable for solving most employee scheduling problems. Hence it has been treated as one of the most used algorithms^31–33^. Plenty of meta-heuristic algorithms have been developed for searching the PARETO solutions and attracted an increasing number of interests^34,35^. The Non-dominated Sorting Genetic Algorithm (NSGA-II^19^) and Multi-objective Evolutionary algorithm based on decomposition (MOEA/D^9^) are two classical MHAs. The PARETO-based rank and crowding distance are proposed to assign the fitness values to each individual, while MOEA/D transforms a multi-objective optimization problem into several single-objective sub-problems, then EA searches the optimal solutions of these sub-problems in parallel^36^. Yuan et al.^37^ proposed an improved Non-dominated Sorting Genetic Algorithm (NSGA-II) algorithm, which presents a novel evaluation function based on ranking level and crowding degree, then the variable proportion-based elitist retention is designed to help generate the optimal solution. However, this method continues to require a large number of generation operations for generating stable and high-quality PARETO solutions. Wang et al.^38^ proposed a hybrid multi-objective evolutionary algorithm based on decomposition (HMOEA/D) to solve the problem. They set a cooperative search operator to generate new solutions, and design an adaptive selection strategy based on the reference point for using the local search operators to enhance exploitation ability.

However, MHAs usually have high time complexity, and due to the randomness of initial conditions and search strategy, they often need to run repeatedly to generate relatively stable results. Our approach adopts the pairwsie-allocated strategy to search for a high-quality schedule, establishes a proficiency average matrix to boost its efficiency, and optimizes the quality of the schedule by flextime strategy.

The third category is the heuristic method, which usually is designed for specific problems. It adopts a series of heuristic strategies to reduce the search space, which aims to speed up the search efficiency and is required to lose part of the result quality. Li et al.^39^ propose a hybrid of iterated greedy and simulated annealing algorithms (IGSA algorithm) to address the flexible scheduling problem, where an improved construction heuristic considering the problem features is proposed to balance the exploration abilities and time complexity. Alzaqebah et al.^40^ present an improved Bee Colony Optimization algorithm for the flexible work time scheduling problem, where a self-adaptive mechanism is used to adaptively select the neighborhood structure to enhance the local intensification capability of the algorithm and to help the algorithm escape from a local optimum. However, this method requires a large number of iteration operations to ensure the feasibility of the generated schedule, which is time-consuming. Khaniyev et al.^41^ address the operating room scheduling problem with the conflicting priorities and preferences of various stakeholders and the inherent uncertainty of surgery duration. They propose a hybrid heuristic algorithm, which defines the objective function in terms of auxiliary functions with a recursive pattern to exactly analyze the optimal surgery duration. However, this method needs too much domain knowledge to build the heuristic models, and high time consumption.

However, heuristic algorithms can be used to solve specific problems, when the problem is changed, the existing algorithm may not be suitable for the new one.

## Conclusion

This paper proposes *FFS*, a polynomial-time solution for soft work time scheduling problems. *FFS* uses the pairwise-allocated strategy to pre-estimate the number of employees for each shift of each day, which effectively shrinks the number of potential assignments, and the proficiency average matrix is established for boosting its efficiency. In addition, it proposes the flextime strategy to decide the soft work time of each employee for each day, which makes the assigned proficiency satisfy the workload of each time period for each day better. Extensive experimental evaluation shows that *FFS* is more effective and efficient than the baselines (i.e. *MILP*,*IPSO* and *MOEAD*), as EXP1-EXP6 shows. Besides, we test the performance of flextime-strategy in improving the effectiveness of *FFS*, as EXP7-EXP8 shows. Hence, *FFS* outperforms the state-of-the-art in our problem.

## Data Availability

The datasets generated and/or analysed during the current study are not publicly available due this study is going on but are available from the corresponding author on reasonable request.
